# Potential Release of Micro- and Nanoplastics from Stormwater Infrastructure

**DOI:** 10.1021/acsestwater.6c00263

**Published:** 2026-06-02

**Authors:** Baqe Doti, Austin Gray, Kyle Strom, Hosein Foroutan

**Affiliations:** † Department of Civil and Environmental Engineering, 1757 Virginia Tech, Blacksburg, Virginia 24061, United States; ‡ Department of Biological Sciences, Virginia Tech, Blacksburg, Virginia 24061, United States

## Abstract

Stormwater systems increasingly rely on polymer-based materials such as polyvinyl chloride (PVC), polyethylene (PE), high-density polyethylene (HDPE), and polypropylene (PP) due to their durability, low cost, and corrosion resistance. However, these materials are susceptible to photochemical, abiotic chemical (e.g., oxidation/chlorination from disinfectants and oxidants), biological (microbial/enzymatic), and mechanical degradation, resulting in the release of micro- and nanoplastics (MNPs) throughout their service life. This perspective critically examines the mechanisms underlying MNP formation in stormwater infrastructure – including ultraviolet (UV)/photoaging, chemical oxidation, hydraulic abrasion, and bed-load interactions – and evaluates laboratory methods used to study these processes. Standardized tools such as the Taber abrasion, Darmstadt rigs, circulating-loop systems, UV weathering, and chemical aging protocols are evaluated for their ability to simulate real-world conditions and quantify plastic particle release. Existing methods primarily quantify material durability but rarely capture or characterize released MNPs, leading to gaps in emission factor development and poor translation of laboratory results to stormwater environments. Analytical techniques such as μ-FTIR, Raman spectroscopy, SEM/EDX, and Py-GC/MS are reviewed for their complementary roles in particle identification and quantification. Key methodological gaps are identified, including inconsistent sampling protocols, limited detection of nanoplastics (NPs), unrealistic hydraulic simulations, and sparse comparisons between recycled and virgin pipe materials. To address these issues, this perspective proposes a hydraulically realistic circulating-loop platform capable of integrating stormwater-like hydraulics with UV and chemical aging, as well as analytical techniques to quantify MNP emissions from pipe materials under environmentally relevant conditions. This integrated framework supports the development of predictive models that link material degradation to MNP release, thereby advancing sustainable infrastructure design and plastic pollution mitigation in water systems.

## Graphical abstract

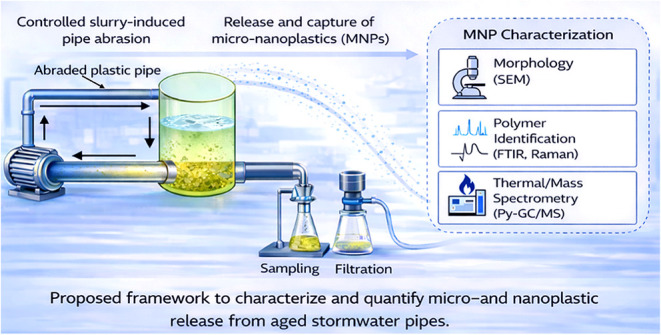

## 1 Introduction

Over the past several decades, traditional materials such as steel and cast iron in drinking water systems and concrete in stormwater systems have been progressively replaced by plastic polymers – including polyvinyl chloride (PVC), polyethylene (PE), high-density polyethylene (HDPE), polypropylene (PP), and glass fiber–reinforced polyester (GRP) resins.^1^ These materials offer advantages such as lower cost, corrosion resistance, and hydraulic performance. However, they are vulnerable to photochemical, thermal, biological, and mechanical degradation mechanisms that can fragment the material into smaller plastic particles.^2,3^ These include microplastics (MPs), typically defined as plastic particles less than 5 mm in size,^4–6^ and even smaller nanoplastics (NPs), with dimensions below 100 nm.^7^ Due to their widespread occurrence, micro- and nanoplastics (MNPs) have gained significant attention as emerging contaminants. Numerous studies have confirmed their presence in stormwater runoff and outflows, where they are typically attributed to roadway wear, atmospheric deposition, and urban litter.^8–10^ Once in aquatic systems, MNPs can be ingested by aquatic organisms, bioaccumulate in food webs, and potentially reach humans through food and drinking water.^11,12^ Health risks associated with MNP exposure include oxidative stress, cytotoxicity, reproductive toxicity, and immune dysfunction.^13,14^

What remains underexplored, however, is the potential role of stormwater infrastructure itself as a source of MNPs. Many stormwater pipes and fittings are manufactured from virgin or recycled plastics that may contain fillers and additives such as calcium carbonate, melamine, silicone compounds, antioxidants, and stabilizers used to tailor stiffness, durability, and processability.^15^ These compositional features, together with prior thermo-mechanical history in recycled materials, may increase defect density, heterogeneity, and susceptibility to oxidation, thereby influencing crack initiation, embrittlement, and particle release during service.^16–19^ In addition to buried pipes, other polymer-based stormwater components – including liners and cured-in-place pipe (CIPP) repair materials, and potentially geotextiles, storage chambers, and catch-basin inserts – may also contribute to MNP emissions, although pipes remain the most extensive and mechanically stressed components of many drainage networks.^20–23^ Abrasive forces from transported sediments and high flow velocities can accelerate surface wear, releasing MNPs. Given the range of physical and chemical stressors stormwater systems experience, it is plausible that pipes are not only conduits but also contributors to MNP pollution. Understanding how pipe material (e.g., virgin versus recycled polymers), hydraulic stress (flow rate), particulate loading (bedload), and environmental exposure influence MNP release is crucial for predicting pollution loads from stormwater infrastructure. This insight will not only constrain MNP loads in stormwater but also provide a basis for designing more advanced pipes composed of materials that reduce MNP release to the environment.

This perspective aims to synthesize representative literature across material science, environmental aging, and stormwater monitoring to highlight why infrastructure-derived MNP release deserves greater attention and to outline a practical research agenda for the field. We therefore focus on the studies most informative for understanding pipe degradation, particle generation, and analytical needs, while using those studies to motivate a stormwater-specific experimental framework. We first examine material, degradation and emission mechanisms. We then summarize stormwater MNP sampling studies that document widespread particle presence, yet do not resolve sources, and examine laboratory approaches for simulating pipe wear and aging. Finally, we identify key methodological gaps and outline research directions for building realistic, predictive frameworks for MNP emission quantification in stormwater systems.

## 2 Infrastructure-Derived MNPs : Materials, Degradation, and Emission Mechanisms

### 2.1 Composition and Distribution of Plastic Materials in Stormwater Infrastructure

Plastics are widely used in stormwater and water-sanitation piping systems due to their favorable mechanical and chemical properties. Common polymers employed in stormwater infrastructure include PVC, HDPE, PE, and PP.^22,24^ Compared to traditional materials such as concrete and metal, plastic pipes are lightweight, cost-effective, flexible, easy to install, and resistant to corrosion, which has contributed to their increasing adoption in drainage and stormwater applications.^25,26^ Beyond pipes, polymeric materials are also used in geotextiles, liners, detention and infiltration structures, and repair systems such as CIPP, expanding the range of stormwater assets that may undergo weathering and particle release.^22^

In the United States, stormwater infrastructure is composed primarily of reinforced concrete, corrugated metal, and plastic pipes, with an estimated total storm sewer length of approximately 3.5 million miles.^27^ However, the specific fraction of this network constructed from plastic materials is not well documented. In contrast, more detailed regional data is available in some countries. In China, the total length of urban drainage pipelines was reported to be approximately 630,200 km, with plastic pipes accounting for about 20% of this network.^28^ In Germany, the total length of stormwater sewer pipelines is estimated at about 125,000 km, with plastics comprising approximately 18.4% in rural areas and 5.5% in urban areas.^29^ In the Netherlands, the combined length of municipal wastewater and stormwater systems is approximately 168,000 km, although the fraction constructed from plastic materials has not been explicitly reported.^30^

A key challenge facing stormwater infrastructure globally is the aging and deterioration of existing pipe networks, where replacement and rehabilitation are often costly and disruptive. To address these challenges, there has been growing interest in the use of recycled plastic pipes as a cost-effective and sustainable alternative. However, recycled polymers can differ from virgin materials in additive package, prior aging history, degree of oxidation, crystallinity, and structural uniformity, all of which may influence long-term durability and fragmentation behavior under stormwater conditions.^15,22^ Establishing the prevalence, material composition, and likely variability of stormwater pipe networks, therefore, provides a critical foundation for evaluating pipe-related MNP release mechanisms discussed in subsequent sections.

### 2.2 Degradation Mechanisms and Emission Pathways

Plastic materials used in stormwater infrastructure gradually undergo a range of transformations that weaken their structure over time. This process, referred to as degradative aging, involves combined thermal, chemical and mechanical alterations that progressively change the material’s properties as shown (Figure 1). In pipe networks, these transformations are often intensified by continuous contact with chemically reactive compounds present in transported water.

**Figure 1:**
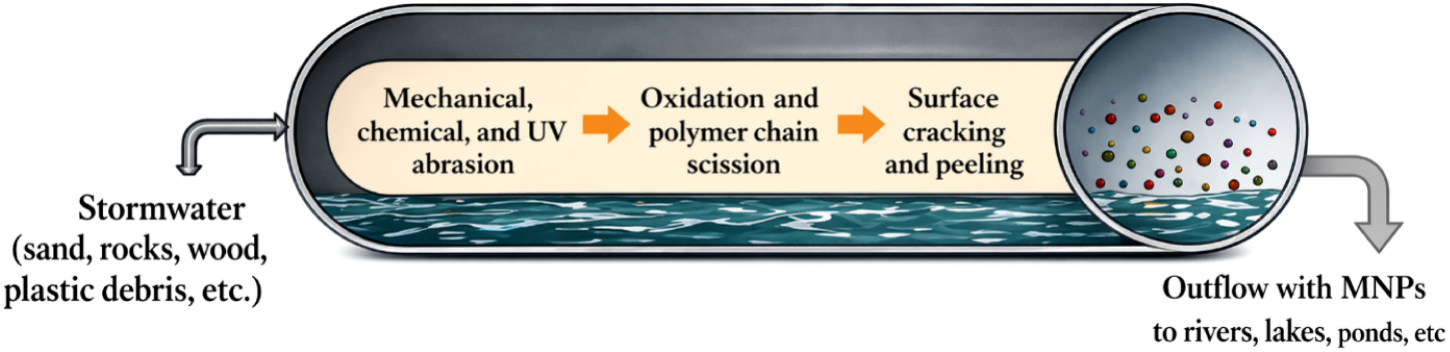
Conceptual schematic illustrating degradation mechanisms and MNP release within stormwater pipes. Mechanical abrasion from suspended solids and bedload materials, together with chemical exposure and polymer aging, promotes oxidation, polymer chain scission, and progressive surface cracking and peeling of the pipe wall. These processes generate MNPs that are transported with stormwater flows toward downstream receiving waters.

Chemical degradation is a major pathway for MNP release. Agents such as disinfectants and pesticides can weaken the structural integrity of plastic pipes. Świetlik and Magnucka,^1^ Whelton et al.^31^ and Zhang et al.^6^ have reported that while plastic pipes resist corrosion, they are vulnerable to chlorine and ozone-based oxidants. Prolonged exposure to such chemicals disrupts the molecular stability of the polymer, diminishing its structural integrity and elasticity.^17^ Over time, the pipe surface becomes brittle and prone to surface flaws such as cracks, scaling, and flaking. Sheng et al.^19^ further demonstrated that exposure to higher chlorine residuals of 1.5 mg/L caused pronounced surface degradation and substantially greater MP release from all tested plastic materials compared to the lower chlorine concentrations of 0.25 mg/L. Accelerated hydrolysis and surface oxidation of polymer chains under alkaline conditions further promote the release of MNPs from plastic pipes. Hammodat et al.^32^ found that deviations from neutral pH – especially in alkaline conditions – enhanced MNP leaching, particularly when free chlorine was also present. The progressive deterioration of the pipe surface through oxidation and cracking eventually leads to the fragmentation of polymer layers, potentially releasing MNP particles into the conveyed water.

Mechanical abrasion from sand and other coarse particles transported in stormwater can significantly accelerate the degradation of plastic pipes. When these abrasive materials repeatedly collide with the pipe surface, they induce oxidation and surface fatigue, which initiate microcracks that progressively penetrate deeper layers of the material, weakening its structure and causing fragmentation.^33^ Shear and tensile stresses exerted by external particles disrupt the molecular chains within polymers, compromising their structural stability and rendering the material more brittle and prone to fracture.^34^ Hydraulic scouring has also been shown to accelerate the detachment of aged polymer fragments. Zhang et al.^15^ investigated the release of MPs from plastic rainwater facilities and found that hydraulic scouring, when combined with surface aging, markedly enhanced MP release across all tested materials, with total amounts ranging from 160 to 1905 items/gram. Similarly, Maliwan and Hu^35^ demonstrated that high-shear, turbulent flow conditions particularly under crossflow filtration modes led to increased MP release from polymeric ultrafiltration membrane systems, linking this to friction between suspended solids and the membrane surface.

Exposure to ultraviolet (UV) radiation from sunlight is another critical factor that accelerates the degradation of plastic pipes. Oxidative reactions triggered by solar UV exposure accelerate plastic degradation, promoting crack formation and fracture propagation that cause the material to become brittle and prone to failure.^36^ UV exposure triggers surface oxidation and molecular chain scission, resulting in the formation of carbonyl (C=O) and hydroxyl (O–H) functional groups that progressively roughen and crack the polymer surface, thereby initiating the release of MP particles.^37^ Hernandez et al.^38^ observed that UV weathering of PS, PP, low-density polyethylene (LDPE), and HDPE under aquatic conditions promoted photo-oxidative degradation, which resulted in surface deterioration and the release of MNPs. Similarly, Cui et al.^39^ reported that prolonged UV irradiation caused photo-oxidative degradation of PP films, with α-PP generating about 71% MPs and β-PP only 25%, indicating that crystalline structure strongly influences the extent of embrittlement and MP release under UV exposure. Supporting these findings, Cai et al.^40^ demonstrated that ultraviolet irradiation caused photo-oxidative degradation of PE, PP, and PS pellets through the formation of hydroxyl and carbonyl groups, leading to surface cracking, flaking, and granular oxidation, with degradation severity highest in air, followed by ultrapure water, and lowest in simulated seawater highlighting the critical role of oxygen availability and UV intensity in governing MNP formation.

Maintenance and repair operations of stormwater and sewer pipes, particularly through cured-in-place pipe (CIPP) technologies, have been increasingly recognized as emerging sources of MNP emissions. Although much of the current literature focuses on volatile and semivolatile organic compound (VOC/SVOC) emissions during these repairs, several recent studies demonstrate that MNPs can also be generated and dispersed during these processes. Morales et al.^20^ revealed that CIPP installations emit substantial quantities of NPs through steam-laden exhaust discharged during resin curing. Their analysis of condensate waste identified colloidal and submicron organic particles (0.5–1 μm) that solidified into NPs upon drying, with an estimated >0.25 tons of NP emissions per project, signifying a considerable yet overlooked pollution pathway. Similarly, Peterson et al.^41^ confirmed that both styrene-based and vinyl ester-based resins used in CIPP processes generate primary MP fragments from cured materials and secondary micrometer and submicrometer particles via aqueous-phase oligomerization reactions. Ra et al.^21^ and Teimouri Sendesi et al.^23^ further documented that steam-cured CIPP installations release complex multiphase plumes composed of organic vapors, condensed resins, and particulates containing polymer residues that can condense or settle in surrounding water bodies if not adequately captured or treated.

Together, these mechanisms illustrate how stormwater infrastructure not only transports MNPs but may also serve as an underappreciated source. Chemical leaching, abrasion, UV degradation, and maintenance emissions all contribute to the transformation of polymer pipelines from passive conduits into active point sources of MNP pollution. Future research should aim to quantify these emissions under field conditions, characterize their particle profiles, and assess how infrastructure design, material choice, and operational practices modulate MNP release potential.

## 3 Detection and Characterization of MNPs in Stormwater

### 3.1 Stormwater Sampling Approaches

Sampling methods for MNPs in stormwater are designed to capture spatial and temporal variability while minimizing contamination. Most studies employ either automatic flow-weighted composite sampling or manual grab sampling during active rainfall events. Containers are typically made of glass or stainless steel, and rigorous sample handling protocols are followed to prevent background contamination. Table 1 summarizes representative stormwater sampling strategies reported in the literature, including system types, sampling volumes, materials used, and key references.

**Table 1:** Stormwater Sampling Approaches

Location	System type	Sampling technique	Sample volume	Container/material used	Ref
Gothenburg, Sweden	Urban stormwater well	Automatic flow-weighted ISCO sampler during rainfall events	2.7–9 L per event	Glass bottles	^42^
Vaughan, Ontario, Canada	Bioretention cell receiving urban runoff	Automatic flow-weighted composite sampling (ISCO 6712) during rainfall >4 cm runoff	0.5–2 L per bottle (composite from 24 bottles)	Polypropylene and HDPE bottles	^43^
Cheonan City, Republic of Korea	Stormwater infiltration trench and highway drainage	Flow-weighted composite sampling during storm events	Not specified	Steel buckets and glass jars	^44^
Gullbergsvass, Gothenburg, Sweden	Urban reconstruction area storm sewer	Automatic ISCO 6712 composite samplers, triggered >5 L/s or >75 mm depth	Not specified (flow-proportional)	Glass bottles	^45^
New Brunswick and Piscataway, NJ, United States	Stormwater outfalls and bioretention basin	Composite sampling using 1 L glass jars collected every 20–40 min during the storm	5 L composite per storm	Glass jars	^46^
Casale sul Sile, Italy	Highway storm drains	Manual grab sampling during rainfall >5 mm	1 L per drain	Glass flasks	^47^
Hong Kong, China	Urban stormwater drainage channels	Grab sampling using stainless steel buckets	Variable	Glass bottles	^48^
Gumi, Republic of Korea	Urban stormwater drain outlet (industrial and residential catchments)	Automatic time-resolved sampling using AS950 automatic water sampler (HACH, US), programmed to collect sample at intervals of 0, 15, 30, 60, 120, 240, and 360 min	6 L per sampling interval	Glass bottles	^49^
Newark, New Jersey, United States	Urban road runoff at drainage gully (residential, commercial, highway)	Manual sheet flow collection at drainage gully during two storm events; samples collected within 30 min after storm start	1 L per location (duplicates collected)	Polyethylene – Linear Low Density collection bag, PYREX glass bottles	^50^
Shanghai, China	Urban surface runoff	Continuous manual sampling using stainless-steel buckets after runoff generation	Not specified	Glass containers	^51^
Pathum Thani Province, Thailand	Urban stormwater runoffs at roadside rainwater draining pits (industrial, transportation, commercial, residential)	Manual first-flush sampling within 30 min of rainfall; duplicated bulk samples with a rinsed metal bucket	2 × 3 L per event	Metal bucket, glass containers	^52^
Daejon, Republic of Korea	Urban stormwater pipeline	Stormwater runoff from urban surfaces collected at the end of the pipeline; 2 L samples at irregular intervals during each rain event (≥5 min early, ≤1 h later)	2 L per sample (per interval)	Field container/material not stated	^53^
Central New Jersey, United States	Urban/suburban stormwater outfalls and a bioretention basin	Discrete grabs on a pole every 10–20 min during rainfall to build composites	10–16 L per storm (two-three composites of 3–8 L)	1 L HDPE bottles	^54^
Bremerton, San Diego and Texas, United States	Stormwater control measures	Automatic samplers: Teledyne ISCO 3700 (with signature flowmeters and rain gauges) or HACH Sigma 900 Max; autotriggered 10 L composites at 5 min intervals	10 L composite per run (as programmed)	10 L glass bottles, vinyl intake tubing	^11^

Despite methodological advancements, these environmental sampling efforts focus solely on characterizing MNP concentrations in runoff or outfalls. As such, they cannot determine whether the detected particles originate from external urban surfaces or from internal infrastructure sources. This limitation underscores the need for studies capable of distinguishing between transported and infrastructure-derived MNPs.

### 3.2 Analytical Techniques for MNP Identification

Following stormwater sampling, it is essential to quantify MNP abundance and chemically characterize the polymers. A range of analytical techniques has been reported in the literature, broadly classified into microscopy, spectroscopy, and thermo-analytical approaches. Across stormwater studies, particle-resolved spectroscopy is often paired with thermal methods to jointly identify and quantify polymer types.

Focal-plane-array Fourier Transform Infrared microspectroscopy (μ-FTIR imaging) provides automated mapping, particle counts, and polymer identification on filters. Pyrolysis gas chromatography mass spectrometry (Py-GC/MS) is widely used to deliver size-independent, polymer-specific mass of particles too small for μ-FTIR characterization. This dual approach has been used in tandem on split subsamples in stormwater studies (e.g., Kirstein et al.;^55^ Liu et al.^56^; Treilles et al.^57^), enabling polymer classification and mass closure for stormwater ponds and rain-event comparisons. A major advantage of Py-GC/MS is that it can quantify total polymer mass even when individual particles are too small, dark, weathered, or irregular for reliable optical identification. However, Py-GC/MS does not provide particle counts, size distributions, or morphology, so it is best interpreted as complementary to spectroscopic particle analysis rather than a replacement for it.^55,58–62^

Where complex or filler-rich matrices hinder FTIR spectral clarity, single-particle FTIR is often supported by attenuated total reflection (ATR) for polymer confirmation, and a scanning electron microscope coupled with an energy-dispersive X-ray detector (SEM/EDX) assists in morphological and elemental verification.^9^ In highway runoff studies, μ-FTIR is typically used for general particle census, while ATR-FTIR (with a germanium tip) and Py-GC/MS focus on black rubber fragments and tire wear.^61,63–65^ Integrated approaches that combine μ-FTIR imaging and Py-GC/MS are increasingly used to close system-level mass balances, particularly for tire wear particles.^60^ Ziajahromi et al.^62^ synthesized findings from multiple studies using μ-FTIR/FTIR-ATR and pyrolysis-based methods, emphasizing the need for rubber-specific thermal markers alongside spectroscopic counts.

The use of Raman mass spectrometry to characterize MNPs in urban stormwater is an additional analytical approach.^66^ The Raman used laser-scanning approaches to create a particle profile that can be paired with known libraries to confirm a polymer match. There are limitations on the size of particles that Raman can characterize; this often depends on the user’s capabilities, creating disparities between particles that are typically larger and can be isolated spectroscopically.

For nanodomain analysis, size-agnostic totals, double-shot Py-GC/MS can quantify common polymers >1 μm and extend down to ∼0.01–1 μm when paired with ultrafiltration and oxidative cleanup.^58,59^ Even so, several analytical challenges remain. Recovery can be strongly affected by pretreatment losses during filtration, digestion, ultrafiltration, or solvent handling; detection limits depend on polymer type and marker selection; and matrix interference from sediment, black carbon, tire wear, dissolved organic matter, and mineral fillers can bias or obscure target signals in complex stormwater samples.^61,67^ These constraints help explain why Py-GC/MS remains underused for stormwater nanoplastics despite its promise for mass-based quantification. For stormwater infrastructure studies, μ-FTIR/ATR-FTIR is typically used for particle screening, with Py-GC/MS applied for polymer-specific mass when needed. In contrast, process-based transport studies (e.g., freeze–thaw) often rely on tracer beads and optical or fluorescence counting rather than polymer-resolved μ-FTIR or Py-GC/MS.^68,69^

Mohamed et al.^70^ used confocal laser scanning microscopy and volumetric imaging to fill the sub-20 μm gap in μ-FTIR throughput, showing close agreement with Py-GC/MS-derived mass estimates for PE and PP fractions. In urban-surface runoff comparisons, Lindfors et al.^67^ reported MPs and tire wear particles using a mixed metric strategy of μ-FTIR-based particle counts for general polymers, and pyrolysis-marker quantification for tire-derived material, explicitly noting that mass outputs depend on the chosen thermal markers and calibration approach.

Together, a standardized toolkit has emerged for stormwater MNPs: μ-FTIR (and ATR-FTIR as needed) and Raman for particle identification and counts, Py-GC/MS for polymer mass and rubber quantification, and SEM/EDX when particle morphology or filler content complicates spectra. The key need moving forward is not simply wider use of advanced instruments, but better integration of these methods through standardized QA/QC, matrix-specific recovery assessment, consistent reporting units, and cross-validation between count-based and mass-based measurements.

### 3.3 Reported MNP Concentrations and Characteristics in Stormwater

Reported MNP concentrations in stormwater vary by several orders of magnitude across climates, drainage settings, and event conditions, suggesting that stormwater networks function both as transport pathways and transient sinks for plastic particles (Figure 2).^71–78^ Rather than pointing to a single representative concentration, the literature consistently shows that reported abundance depends strongly on rainfall intensity, antecedent surface loading, catchment land use, sampling design, and the lower particle-size cutoff used in each study.

**Figure 2:**
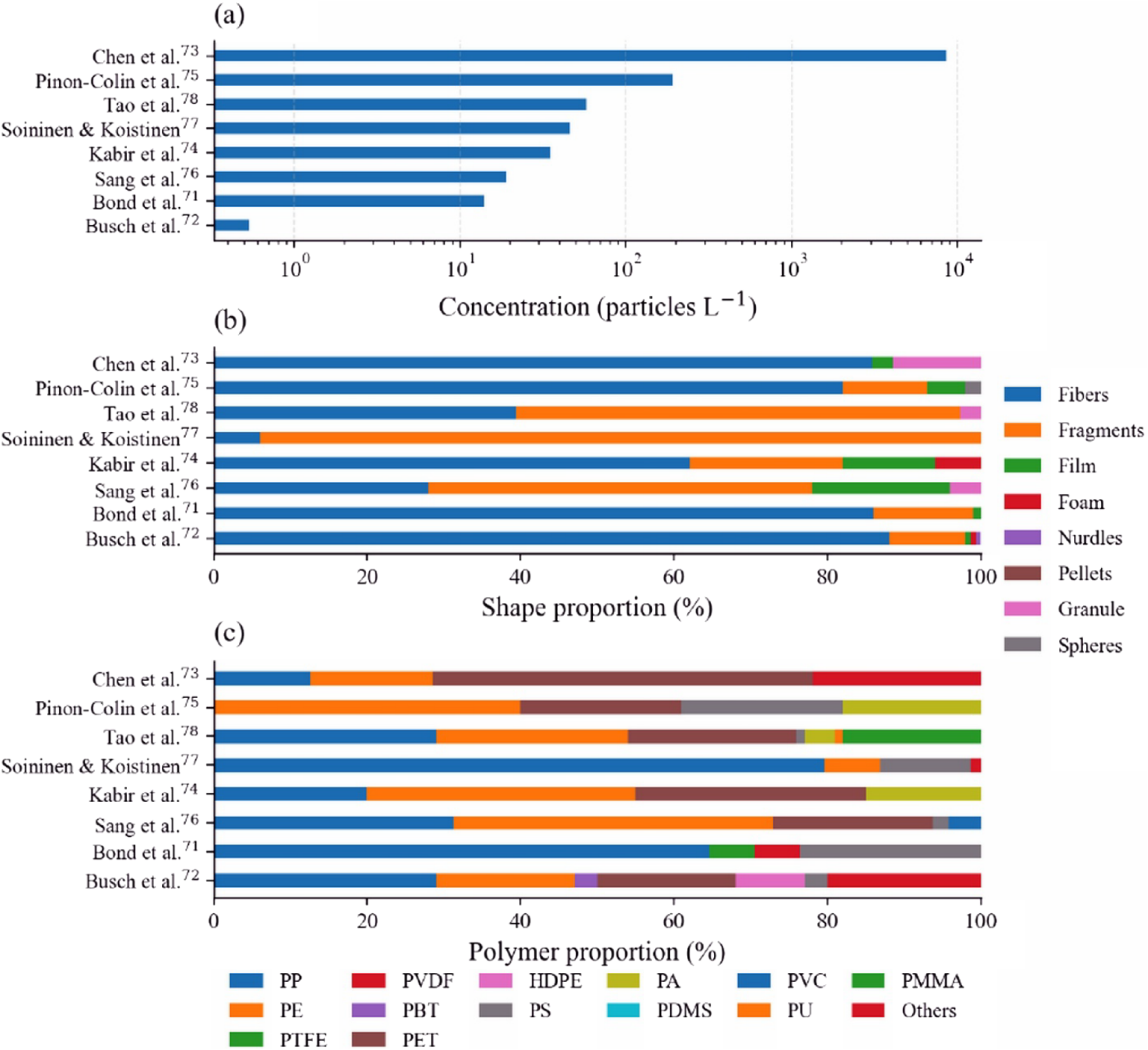
Reported concentrations and characteristics of MNPs in stormwater and urban runoff from selected peer-reviewed studies. (a) MNP number concentrations (particles L^–1^) reported across studies, plotted on a logarithmic scale to capture the several-order-of-magnitude variability observed in stormwater systems. (b) Relative contributions of MNP shapes expressed as percentages within each study. (c) Polymer composition of detected MNPs, showing the proportional distribution of commonly reported polymers.

Across temperate, subtropical, and highly urbanized environments, several consistent patterns emerge. Stormwater samples are typically dominated by particles smaller than 1 mm, with concentrations increasing substantially when finer fractions are included.^54,79,80^ In terms of morphology, fibers and fragments are most frequently reported, although black rubbery particles and tire-wear-associated fragments become more prominent in traffic-influenced systems.^62,63,74,81^ Polymer composition is also broadly consistent, with PE and PP frequently dominant, while PET, PS, PVC, PA, PU, HDPE, and fluorinated polymers appear as secondary contributors depending on land use and analytical method.^54,62,67,71,79^

A second recurring observation is that hydrology and source setting are as important as geography. Road runoff, combined sewer overflows, athletic-field drainage, and first-flush samples often yield higher concentrations than more buffered systems, reflecting the strong influence of storm intensity, traffic, surface accumulation, and drainage connectivity on particle mobilization.^49,51,72,73,82^ While sedimentation structures and treatment trains can partially retain MNPs, they also act as temporary reservoirs that may be remobilized during subsequent events.^43,60,83,84^ This dynamic helps explain the substantial variability observed even among studies conducted within similar regions.

The stormwater occurrence literature is therefore most informative when interpreted comparatively rather than study by study. Across systems, small particles, fragment- and fiber-dominated morphologies, and PE/PP-rich polymer profiles are consistently observed, while tire-derived particles are especially important in traffic-dominated runoff. At the same time, these studies rarely resolve whether detected particles originate from urban surface sources, atmospheric deposition, or in situ generation within the drainage infrastructure itself. This unresolved source apportionment remains a central challenge and motivates the need for infrastructure-focused experiments and emission factor development.

In pipeline systems, Tao et al.^78^ reported that MPs persist along a long-distance raw-water pipeline, with concentrations in the single- to low double-digit items-per-liter range that vary by location and season. Most particles were 10–100 μm in size and predominantly fragments, with polymers including poly­(methyl methacrylate) (PMMA), PE, and PP. These findings suggest that both upstream inputs and infrastructure-related processes can contribute to observed concentrations, reinforcing the broader point that occurrence data alone are insufficient to separate background transport from infrastructure-derived release.

## 4 Laboratory Methods for Pipe Degradation and MNP Release

### 4.1 Taber and Darmstadt-Type Abrasion Tests

Most existing work on mechanical degradation of polymeric materials relevant to water infrastructure has been carried out using standardized abrasion rigs, with Taber abrasion tests (Figure 3a) dominant for flat coated panels and Darmstadt-type erosion tests (Figure 3b) more common for pipes and flowlines. In the coating literature, Cambruzzi et al.^85^ used a three-body Taber configuration, where rubber wheels carried abrasive sands across polyester powder coatings on steel, and evaluated degradation via mass loss, thickness reduction, and electrochemical impedance spectroscopy (EIS). They found that fine particles caused gradual loss of protective properties, whereas coarse grains produced large defects and rapid failure. Crucially, they also showed that mass loss alone underestimated loss of barrier function compared to EIS-derived pore resistance. Building on this, Rossi et al.^86^ modified the standard Taber setup to allow in situ electrochemical monitoring of organic coatings under abrasion by a sand–electrolyte slurry, enabling real-time measurement of the decrease in corrosion protection during the test. In a subsequent study, Rossi et al.^87^ used the same modified Taber setup with natural sands of differing grain size, morphology and mineralogy, and showed that the reduction in barrier effect depended strongly on sand characteristics where larger, harder grains and lower calcite content increased abrasiveness, while rounded desert sands were less aggressive than sharp-edged sands. These studies established the Taber-slurry tests as a valuable tool to link mechanical abrasion to functional coating loss, but the response is still expressed in terms of coating-level metrics (pore resistance, thickness, mass loss) rather than the properties of abraded particles.

**Figure 3:**
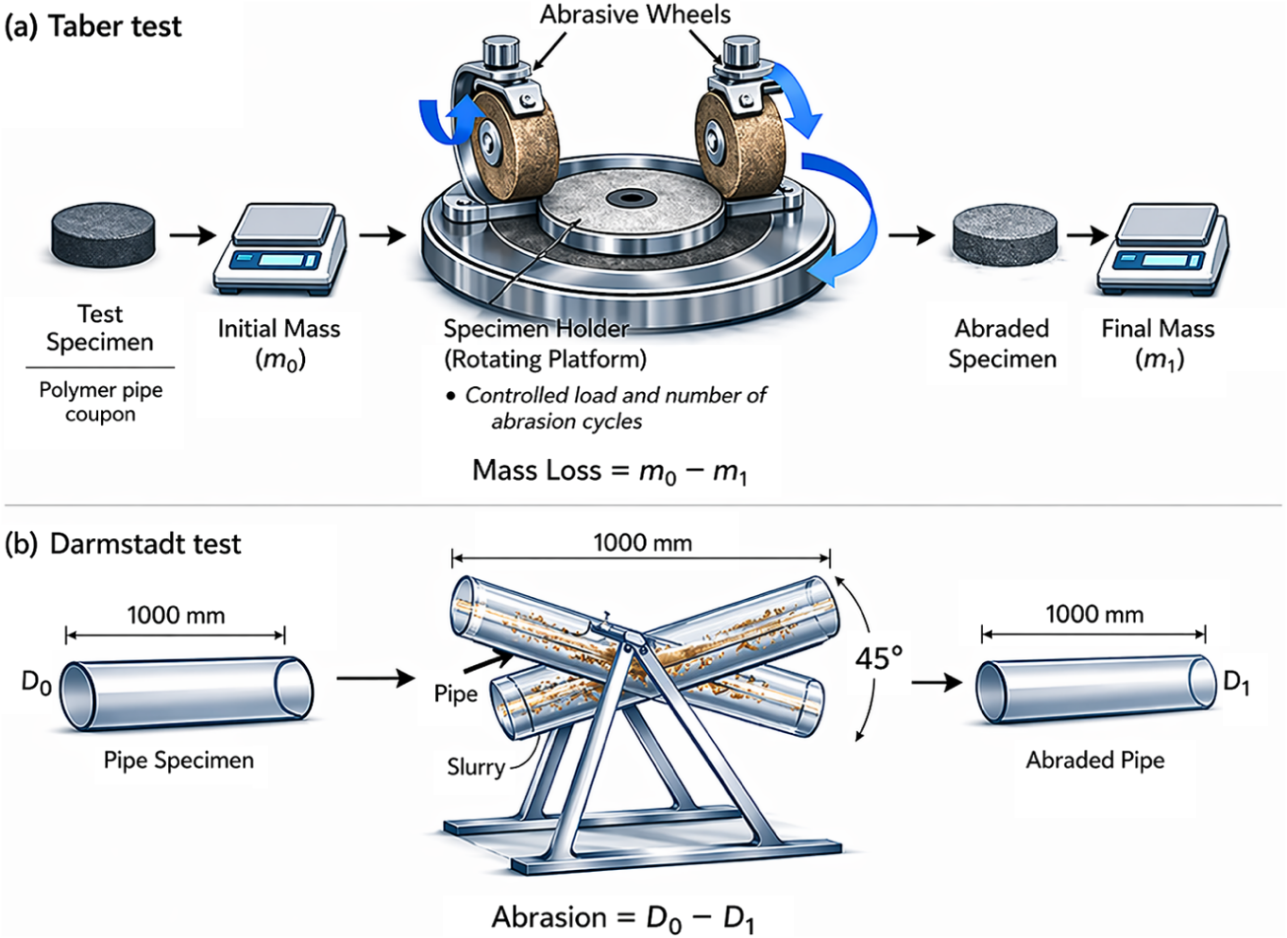
Traditional laboratory methods used to quantify abrasion of plastic pipes and pipe materials. (a) Taber abrasion test, in which a polymer pipe coupon is subjected to controlled mechanical wear using rotating abrading wheels under a specified load and number of cycles, with abrasion quantified by mass loss (m_0_ – m_1_). (b) Darmstadt abrasion test, where full-length pipe specimens are mounted at a fixed inclination (typically 45°) and exposed to slurry flow containing abrasive particles, with abrasion quantified by the reduction in pipe wall thickness or diameter (D_0_ – D_1_) after testing.

Rossi et al.^88^ later extended this Taber-based framework to include the role of chemical environment and abrasion. In their coil-coating study, a slurry of Al_2_O_3_ sand and organic solutions was used in a modified Taber test to examine how aggressive ions, pH and solvents altered abrasion damage and protective properties of prepainted steel panels. They showed that organic solvents could strongly interact with the paint matrix, leading to cracking and local loss of protection under a given mechanical load. In a related study on organic coatings, Rossi et al.^89^ focused on “mar” damage (gloss loss). Taber abrasion with different abrasive pastes was used to quantify changes in gloss at 20° and link these to microscopic scratch morphology, demonstrating that increasing abrasive grain size led to more severe aesthetic degradation even when corrosion protection remained acceptable. Scrinzi et al.^90^ extended this aesthetic-and-durability perspective to nanosilica-modified automotive clear coats, combining Taber tests and falling-abrasive tests with artificial weathering. They found that nanosilica and higher cross-link density improved mar and erosion resistance, and that thermal treatment after slight abrasion allowed partial gloss recovery through coating reflow, while UVA exposure slightly enhanced mar resistance via additional cross-linking. Together, these studies show how Taber-type tests can be integrated with optical (gloss, color), chemical (FTIR) and electrochemical measurements to build a multidimensional picture of coating degradation under combined mechanical and environmental stresses, but their outputs remain focused on surface appearance and corrosion performance rather than quantifying or characterizing the abraded polymer fragments.

Beyond conventional Taber rigs, several authors have explored micro- and nanoscale mechanical testing to understand how intrinsic material properties control abrasion response. Yahyaei and Mohseni^91^ prepared sol–gel-derived, UV-cured nanocomposite films on polycarbonate and used nanoindentation, nano scratch and Taber tests to link hardness and elastic modulus to scratch and abrasion resistance. They showed that higher inorganic content and optimized silane composition increased hardness and elastic modulus, which in turn enhanced scratch resistance and allowed better elastic recovery during sliding contact; interestingly, they found that moderate rather than maximum hardness gave the best abrasion performance, highlighting the importance of balancing stiffness and toughness. Liu et al.^92^ studied SEBS–PP elastomers, finding that continuous PP phases and good interfacial adhesion improved wear resistance, emphasizing how microstructural tailoring can mitigate surface loss under flow. Still, these studies treat wear as a bulk material loss rather than a source of MNPs.

Darmstadt-type tests better reflect pipe conditions by exposing actual pipe segments or liners to flowing slurries. Stabik et al.^93^ used an erosion testing stand to assess the resistance of plastic pipes to solid–liquid erosion, exposing pipe segments to flowing slurries of abrasive particles and quantifying mass loss and wall thinning as a function of exposure time and operating parameters. Rubin et al.^94^ employed a dedicated bed-load erosion rig to study how moving sediments erode rigid pipes made of homogeneous materials, mapping erosion patterns, wall thinning and subsequent effects on structural strength under pressure. Their work highlighted that erosion is often spatially heterogeneous, controlled by secondary flow structures and bed-form development, and that localized thinning can significantly reduce pipe burst pressure long before uniform wall loss would predict failure. Kovriga et al.^95^ focused on developing PE pipes with enhanced wear resistance for pulp hydro-transportation, using an accelerated wear test where pipes were exposed to abrasive pulp suspensions and wear was quantified via wall thickness measurements and relative wear rates; they then linked wear performance to pipe formulation (polymer grade, stabilizers) and operating conditions. Duarte-Poveda et al.^96^ evaluated HDPE liners as alternatives to metallic materials in corrosive, erosive flowlines, using pilot-scale flow-loop or drum-type tests with sand-laden fluids to demonstrate that HDPE linings markedly reduced corrosion wear compared to unlined steel, again using wall thickness and visual inspection as primary metrics. Collectively, these studies treat erosion as a structural integrity problem and quantify performance in terms of mass loss, thickness reduction or residual burst strength, which is appropriate for design and asset management but does not explicitly consider the fate of the eroded polymer as MNPs.

Some recent work on thick organic offshore coatings further blurs the line between Taber-type and Darmstadt-type methods by using rotating wheels or jet-impingement rigs with abrasive slurries to simulate real offshore conditions. Momber et al.^97^ investigated how polymer hardness affects abrasive wear resistance of thick offshore coatings by subjecting coated steel panels to slurry abrasion and showed that higher hardness generally improved wear resistance but that the relationship was not strictly linear, with coating formulation and microstructure also playing important roles. Although such tests often involve wet, particle-laden contact like what would occur in storm sewers and culverts, the particle phase is treated as a generic abrasive agent rather than an object of interest.

Across these Taber and Darmstadt-type studies, several consistent limitations emerge when viewed from an MNP generation perspective. First, the abraded material is almost always quantified indirectly via mass loss, thickness reduction, gloss change or electrochemical loss of barrier function, rather than by direct counting, sizing or polymer identification of the detached particles. As a result, there is essentially no information on the number concentration, size distribution, shape or polymer crystallinity/aging state of the particles generated by these abrasion protocols, despite these being key parameters needed to define MNP emissions and their ecotoxicological effects. Second, the vast majority of Taber-based works are conducted on flat coated panels (coil coatings, powder coatings, automotive clear coats) under relatively idealized contact conditions, which differ fundamentally from the internal hydraulics of stormwater pipes, where particles are mobilized by gravity-driven or pressurized flows, interact with pipe joints and fittings, and experience highly variable shear and turbulence. Third, even in pipe-like geometries, tests focus on structural performance rather than environmental release. These tests do not sample or analyze the carrier fluid or sediment fractions to determine how much plastic is being exported from the system, and they do not attempt to link local wall thinning or erosion patterns to an MNP mass or number flux per meter of pipe. No study among those reviewed here explicitly frames the eroded polymer as MNPs, nor do they apply MNP-specific analytical tools such as μ-FTIR, Raman, Py-GC/MS or particle imaging (described in Section 3) to the effluent or bed material. Finally, none of these Taber or Darmstadt-type studies are designed to replicate stormwater-specific conditions – intermittent flow, short high-shear bursts, mixed bed loads (e.g., tire wear, road dust, organics), or the chemical composition of stormwater. Instead, mechanical and environmental variables are chosen primarily to accelerate coating or pipe wear for structural design purposes.

### 4.2 Circulating-Loop Abrasion Systems

Circulating-loop and slurry-recirculation systems offer hydrodynamically realistic laboratory representations of erosive wear in pipelines, as they maintain continuous slurry motion under controlled velocities, particle loadings, and turbulence structures. Across the available literature, these systems have been used to understand how flow regime, sediment characteristics, and pipe material properties interact to produce erosion under steady or transient slurry transport. Although primarily used for metallic or abrasion-resistant materials, they yield valuable insights into how flow regime, particle morphology, and wall properties govern material loss.

Huang et al.^98^ developed a detailed erosion model for horizontal slurry flow, linking turbulence-induced particle impacts, particle size and shape, material hardness, and wall shear stresses to erosion rate. Their phenomenological formulation demonstrated that the velocity exponent in the erosion relationship can range from approximately 2.0 to 3.575 depending on impact conditions, particle morphology, and slurry dynamics, and that coarse, angular particles tend to generate disproportionately severe wear. These findings align with other mechanistic studies such as Xie et al.,^99^ who used recirculating test rigs to compare the abrasion performance of different materials and observed a strong dependence of wear rate on particle hardness, angularity, and turbulent intensity. These studies emphasize that erosive wear in flowing slurries is governed by a combination of particle kinematics and wall material properties, controlled primarily by the turbulence and velocity field of the carrier fluid.

Experimental studies have confirmed that nonuniform flow fields create spatially variable wear. Graham et al.^100^ and Zhou et al.^101^ showed that secondary currents, swirling flows, and localized accelerations intensify erosion at pipe inverts and bends. Qiu et al.^102^ combined experiments and simulations to show how wear-induced surface roughness alters flow resistance and erosion patterns, creating feedback loops. Sarker et al.,^103^ using the Toroid Wear Tester, showed that only specific particle sizes and flow velocities produce realistic sliding beds, emphasizing the role of particle–wall dynamics.

Industrial-scale slurry transport studies further demonstrate how wear evolves under dense, coarse slurries. Calderón-Hernández et al.^104^ found that wear in iron ore slurry transport pipelines increases substantially with pumping time due to both particle degradation and progressive pipe-wall thinning, with the most intense damage occurring where coarse ore particles interact with elbows and reducers. Chung et al.^105^ documented erosion–corrosion behavior in slurry pipelines, showing that mechanical erosion continuously exposes fresh metal surface, while corrosion processes weaken the material and accelerate subsequent abrasive loss. Walker and Hambe^106^ demonstrated that particle shape exerts a strong influence on slurry-induced wear, with blocky or angular particles generating far higher wear rates compared with spherical particles of the same size and mineral composition. Wang et al.,^107^ investigated cemented paste backfill transport, showed that wear rate increases nonlinearly with aggregate concentration, slurry rheology, and flow velocity, with abrasion and erosion mechanisms accounting for more than 70% of total material removal. Their study emphasizes the role of particle–particle interactions and collision frequency in determining wear progression under dense slurries. Laboratory work by Rendón and Olsson^108^ similarly concluded that coarse and angular particles intensify wear in abrasion-resistant steels and that ductile and brittle materials exhibit fundamentally different wear responses under identical slurry conditions. Collectively, these studies illustrate how sliding and microimpact erosion evolve across a range of industrially relevant slurry environments.

Across the complete body of circulating loop and slurry-recirculation research reviewed for our perspective, several overarching limitations emerge with respect to MNP research in stormwater systems. All studies quantify erosion exclusively through macroscopic metrics such as gravimetric mass loss, wall-thickness reduction, surface profilometry, or changes in structural capacity. None collect or analyze the eroded debris to determine whether the detached material contains MNPs, nor do they attempt to characterize particle size distributions, morphologies, or material composition. The materials tested typically steel, abrasion-resistant alloys, or white iron differ fundamentally from polymer-based stormwater pipes, whose lower hardness, ductility, and viscoelastic behavior would yield qualitatively different erosion and fragmentation patterns under similar flow conditions. Additionally, slurry conditions used in these studies include high solids concentrations, coarse mining aggregates, and chemically aggressive media that bear little resemblance to urban stormwater, which exhibits variable sediment loads, organic contaminants, and environmental aging effects on pipe materials. While circulating-loop systems offer valuable insights into hydraulic abrasion mechanisms, they do not provide empirical data on MNP release, polymer fragmentation, or the environmental fate of particles generated from pipe materials under stormwater relevant conditions.

### 4.3 UV and Photodegradation Experiments

UV radiation drives photo-oxidative degradation of polymers, leading to chain scission, embrittlement, and MNP release. Across the studies most relevant to stormwater materials, UV aging is consistently associated with the formation of oxygenated functional groups, increased brittleness, surface cracking, and greater susceptibility to fragmentation under subsequent mechanical stress. Although the tested materials and exposure conditions vary, the broader implication is clear: photoaging can precondition polymer pipes and related stormwater components for accelerated MNP release once hydraulic abrasion begins.

Evidence from LDPE and HDPE studies illustrates these common patterns. Doğan^109^ documented progressive oxidation and formation of carbonyl-, hydroxyl-, ether-, and carboxylate-containing groups during LDPE irradiation, while Nguyen et al.^18^ found that both virgin and recycled HDPE drainage pipes lost tensile strength and elongation with UV exposure, with recycled HDPE degrading more rapidly. Similar sunlight-driven embrittlement, whitening, cracking, and mechanical weakening have been reported for HDPE and LDPE in other infrastructure-relevant polymer systems.^110,111^ These results support the idea that prior UV history may strongly influence the abrasion response of stormwater pipes, especially where recycled material is used.

Broader fragmentation studies further show that UV aging can directly increase the generation of small MPs and NPs. Huang and Wang^112^ reported increased production of very small MPs and NPs after photoaging, while Zjacić et al.^113^ showed that photoaged PP fragmented into smaller particles and released more associated metals than pristine PP under the same mechanical treatment. Dai et al.^114^ similarly identified UV irradiation and abrasion as the dominant drivers of freshwater photoaging, highlighting their coupled role in oxidation and crack propagation.

These observations provide strong mechanistic support for including UV/photoaging in stormwater pipe studies, but the current literature remains limited in ways that matter for this perspective. Most experiments rely on static chambers, simplified water media, or exposure conditions that do not capture the intermittent shading, burial, wetting–drying cycles, and sediment-laden flows typical of stormwater networks. More importantly, very few studies quantify particles released specifically from UV-aged drainage pipes under realistic hydraulic conditions. The key research need is therefore not more proof that UV damages polymers, but integrated experiments that connect photoaging history to stormwater-relevant MNP emission rates and particle characteristics.

### 4.4 Chemical Aging Protocols

Chemical aging of plastic pipes in stormwater systems can arise from prolonged exposure to oxidizing and reactive environments associated with urban runoff, including dissolved oxygen, ozone formed during photochemical smog events, peroxides, road-derived salts, organic acids, hydrocarbons, metal-rich particulates, and intermittent disinfectant exposure in mixed or reused water systems. Although most existing evidence comes from drinking-water contexts, the underlying mechanisms – antioxidant depletion, oxidation of polymer chains, embrittlement, crystallinity changes, microcrack initiation, additive leaching, and in some cases direct MNP release – are highly relevant to stormwater infrastructure.

Studies of disinfectant-exposed polymer pipes establish a coherent mechanistic foundation. Khan et al.^17^ showed that chlorine dioxide and sodium hypochlorite markedly decreased antioxidant levels, reduced ductility, and increased embrittlement across common pipe materials, while Vertova et al.^115^ likewise documented deterioration of plastic pipes in a semiclosed circulation system. Zhang et al.^6^ then demonstrated direct MP release from ozone-exposed pipe materials, linking strong oxidation to chain scission and enhanced particle detachment. Related work has also shown that chemically aged plastics can release leachates that alter microbial communities or add toxicologically relevant dissolved compounds to water.^14,16,116,117^

The strongest insight from these studies is that oxidants do more than weaken bulk material properties: they can create brittle surface layers, consume protective additives, alter crystallinity, and facilitate subsequent particle release. Yu et al.^118^ showed that chlorine dioxide penetrated deeply into polyethylene pipe walls and formed a brittle oxidized layer, while Fujii et al.^119^ found that pressure amplified chlorine-driven degradation of polybutylene pipes. Sheng et al.^19^ further reported that realistic residual chlorine concentrations increased concentrations of 1–40 μm MPs in stagnant water and promoted visible surface degradation, with PVC releasing more particles than PE.

For stormwater applications, however, the literature remains only partially transferable. Most studies use static or simplified exposure conditions and then assess degradation through antioxidant depletion, tensile loss, crystallinity change, or surface morphology, rather than directly characterizing released particles in flowing, sediment-bearing water. Stormwater systems also differ from drinking-water systems in pH variability, dissolved organic matter, particulate loading, UV exposure, and redox cycling. These differences suggest that chemical aging in stormwater may be more heterogeneous and more tightly coupled to abrasion than existing protocols currently capture.

Accordingly, the main value of the chemical-aging literature for this perspective is not as a complete analogue of stormwater conditions, but as evidence that oxidative exposure history is likely to be an important control on infrastructure-derived MNP emissions. The next step is to integrate stormwater-relevant chemistry with flow, sediment abrasion, and direct MNP analytics so that chemical preconditioning can be translated into particle release rates and emission factors, rather than only material durability metrics.

### 4.5 Toward Stormwater-Relevant MNP Emission Frameworks

Taken together, the abrasion and degradation studies reviewed here show that Taber and Darmstadt tests, circulating-loop rigs, UV/photoaging protocols, and chemical-aging experiments each illuminate important aspects of pipe material performance, but they were developed primarily as durability tools rather than as methods for quantifying MNP emissions under stormwater-relevant conditions. Across these approaches, degradation is usually inferred from bulk or surface-level metrics – mass loss, wall-thickness reduction, gloss change, antioxidant depletion, crystallinity increase, or mechanical-property decline – while the detached particles themselves are rarely collected, counted, sized, or chemically identified.

None of the existing approaches simultaneously reproduce the unsteady hydraulics, mixed bed load, recycled-versus-virgin material differences, and combined UV–chemical aging that characterize stormwater systems, and almost none translates abrasion or aging into emission factors per meter of pipe. A hydraulically realistic circulating-loop system (Figure 4), therefore, remains the most promising platform for this perspective because it can integrate controlled stormwater-like hydrographs, bed load, and road-derived particulates, and pre- or coexposure of pipes to UV/photo and oxidant chemistries, while coupling those experiments to direct MNP analytics and predictive model development. Such a setup can simultaneously mimic stormwater conditions and directly quantify the number, size distribution, morphology, polymer type and surface properties of MNPs released from recycled and virgin stormwater pipes.

**Figure 4:**
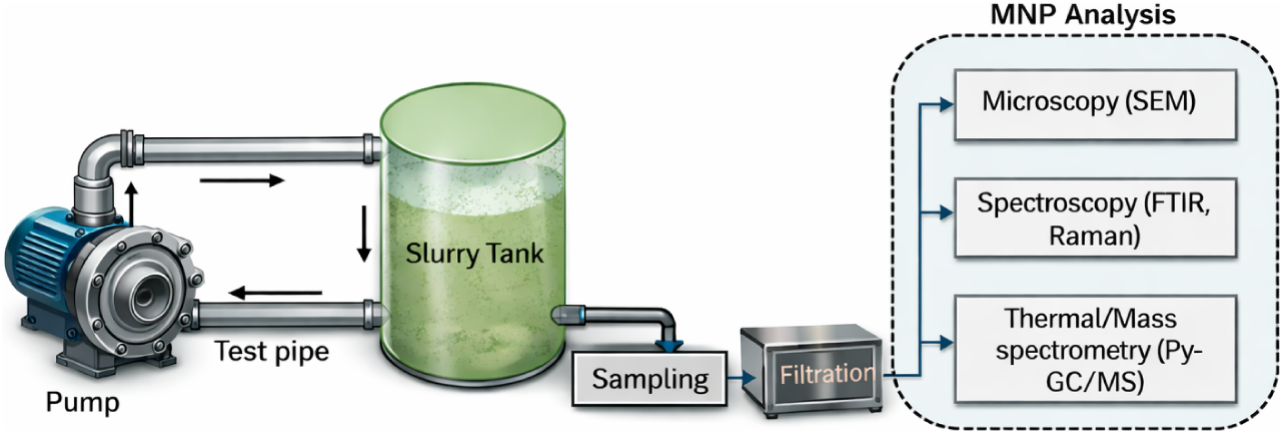
Schematic illustration of the proposed closed-loop circulating system for investigating MNP release from stormwater pipes under hydraulically realistic conditions.

## 5 Knowledge Gaps and Future Research Needs

Although substantial progress has been made in identifying the occurrence and behavior of MNPs within aquatic environments, important uncertainties remain regarding their release, transport, and transformation in stormwater systems. From a stormwater-infrastructure perspective, the most pressing needs are not simply more occurrence data, but validated protocols, integrated experiments, and scalable predictive tools that can connect polymer degradation to real-world emissions. The major gaps and corresponding research priorities are summarized below.

i Lack of standardized sampling and analytical methodologies: Current studies employ inconsistent sampling and analytical approaches, with wide variations in mesh or filter sizes, sampling volumes, reporting units, and contamination-control procedures, resulting in large discrepancies in MNP concentration and characterization data. Future work should move toward validated stormwater-specific protocols that define sample volumes, sieve and filter cutoffs, field and laboratory blanks, polymer-confirmation workflows, and harmonized reporting in both count-based and mass-based units. Interlaboratory comparisons are especially needed to evaluate reproducibility across diverse stormwater matrices.
ii Limited application of pyrolysis–gas chromatography–mass spectrometry (Py-GC/MS) for NP detection: Py-GC/MS remains underused in stormwater studies, particularly for small particles and NPs that fall below the practical limits of optical methods. Its value lies in providing polymer-specific mass independent of particle visibility, but detection limits, recovery losses, and matrix interference from sediment, black carbon, dissolved organic matter, and fillers still constrain routine use. Future investigations should therefore focus on matrix-specific recovery testing, improved cleanup workflows, and deliberate pairing of Py-GC/MS with μ-FTIR, Raman, or imaging methods to improve polymer mass balance across the full particle-size spectrum.
iii Insufficient translation of laboratory data to environmental scales: Most laboratory experiments quantifying MNP release from pipe materials have not been effectively scaled to real stormwater systems. A priority for future work is development of emission-factor frameworks that express release as a function of pipe length or wetted area and then link those rates to rainfall intensity, hydrograph shape, sediment load, pipe age, and network extent. Such approaches would allow laboratory measurements to feed directly into drainage-network and watershed-scale models of cumulative MNP loading.
iv Limited assessment of biofilm effects on MNP release and environmental fate: Pipe materials used in laboratory studies are not perpetually clean surfaces. Biofilms can colonize inner pipe walls, potentially accelerating degradation. As MNPs are released, they may carry biofilms on their surfaces, altering buoyancy and promoting trapping in sediments rather than downstream transport after release into surface waters. Future work should grow representative biofilms on pipe sections before testing and compare conditions with vs without biofilm under realistic flow, sediment, and water chemistry. Measure changes in MNP release rate and physicochemical characteristics, and conduct settling/transport tests to quantify aggregation and deposition for modeling.
v Inadequate simulation of realistic hydraulic and bed-load conditions: Existing abrasion studies often use simplified mechanical setups that fail to replicate the dynamic shear stress, turbulence, and sediment-particle interactions typical of stormwater systems. This limitation may underestimate actual MNP release rates under field conditions. Developing advanced recirculating pipe rigs capable of simulating unidirectional flow, variable sediment concentrations, and pipe inclination angles would bridge this gap. Such systems should operate under velocity ranges representative of storm events and allow in situ monitoring of MNP generation, pipe surface degradation, and associated physicochemical changes in the circulating water.
vi Incomplete quantification of MNP release during pipe abrasion: Most existing studies examining pipe abrasion have primarily focused on measuring changes in pipe-wall thickness or surface roughness to assess material durability, rather than directly quantifying the MNPs released during the abrasion process. This approach overlooks the crucial link between physical pipe wear and the generation of secondary plastic contaminants, leaving a significant gap in understanding how mechanical degradation translates into pollutant release. Future investigations should move beyond structural characterization and incorporate direct MNP quantification under controlled abrasion conditions.
vii Limited understanding of recycled versus virgin polymer behavior: Few studies have compared the MNP-release behavior of recycled and virgin polymer pipes under identical flow and chemical conditions. Because recycled materials may contain additives, residual stresses, or structural heterogeneity, they might degrade more rapidly than virgin counterparts. Systematic comparative studies are needed to quantify the influence of material origin on MNP release and mechanical wear. Conducting controlled abrasion tests with virgin and recycled HDPE, PVC, PE, and PP pipes, followed by morphological and chemical characterization of the released particles, provide insights into long-term durability and environmental trade-offs of using recycled materials in stormwater infrastructure.
viii Lack of integrated predictive and monitoring frameworks: There is currently no comprehensive framework that links laboratory abrasion experiments, field monitoring, and predictive modeling to estimate MNP emissions from stormwater systems over time. Future efforts should couple laboratory-derived emission factors with hydraulic and watershed models and then test those predictions against field observations from outfalls, treatment structures, and representative pipe networks. Such integration would help identify emission hotspots and support prioritization of monitoring and mitigation efforts.

Addressing these knowledge gaps will require a coordinated framework that combines standardized stormwater sampling protocols, realistic hydraulic and aging experiments, and analytical workflows capable of capturing both particle counts and polymer mass. Just as importantly, the resulting data should be translated into emission factors and predictive tools that are useful for infrastructure design, material procurement, maintenance planning, and policy development. In that sense, improving understanding of infrastructure-derived MNPs is not only a scientific need, but also a practical step toward more sustainable and publicly defensible stormwater management.

## 6 Conclusions

Plastic-based stormwater infrastructure represents a plausible and understudied source of MNP pollution. This perspective highlights that pipe materials can undergo significant mechanical, photochemical, and chemical degradation over their service life, with abrasion from transported sediments, UV/photoaging, and oxidative chemical exposure collectively increasing the likelihood of fragment release. Existing laboratory methods have generated valuable insight into polymer durability, but they seldom quantify the detached particles themselves, while most environmental occurrence studies document stormwater MNPs without resolving whether infrastructure is a direct source.

Progress in this area will depend on reframing durability testing into stormwater-relevant emission science. A hydraulically realistic circulating-loop platform that combines unsteady flow, sediment transport, recycled-versus-virgin materials, and controlled UV/chemical aging offers the strongest path forward because it can generate direct MNP release data under conditions that resemble actual drainage networks. When coupled with integrated analytics such as μ-FTIR/Raman and Py-GC/MS, such experiments can produce the emission factors needed for predictive modeling and for better decisions on infrastructure design, material selection, monitoring priorities, and mitigation of plastic pollution in urban water systems.
